# Leveraging the Translational Science Benefits Model to plan and measure early impact in the heart failure polypill implementation trial in Sri Lanka

**DOI:** 10.1017/cts.2026.10699

**Published:** 2026-02-06

**Authors:** Adam Hively, Anna La Manna, Ella Clark, Douglas Luke, Asita de Silva, Mansi Agarwal, Mark D. Huffman, Abdul Salam, Anubha Agarwal, Emmanuel K. Tetteh

**Affiliations:** 1 Division of Cardiology, School of Medicine, Washington University, St. Louis, USA; 2 Center for Public Health Systems Science, School of Public Health, Washington University, St. Louis, USA; 3 Department of Pharmacology, Faculty of Medicine, University of Kelaniya, Sri Lanka; 4 Institute for Informatics, Data Science, and Biostatistics, School of Medicine, Washington University, St. Louis, MO, USA; 5 The George Institute for Global Health, University of New South Wales, Sydney, Australia; 6 The George Institute for Global Health, Hyderabad, India; 7 Prasanna School of Public Health, Manipal Academy of Higher Education, Manipal, India

Demonstrating the impact of research is increasingly important in today’s funding and accountability climate. Funders, policymakers, and the public are asking not only what science is being conducted, but what difference it makes [1,2]. While substantial impact, especially when it involves explicit, definitive clinical outcomes, often requires years to become visible, meaningful change can be seen earlier. This work emphasizes how early integration of the Translational Science Benefits Model (TSBM) enables research teams to identify and communicate near-term benefits during project implementation, rather than waiting until study completion. Intermediate impacts, such as improvements in clinical practice, stakeholder engagement, or public awareness, can alter the trajectory of research [3,4]. Communicating these early and projected benefits offers the scientific community opportunities for dialogue, collaboration, and refinement of strategies. Yet, understanding how to demonstrate impact and when to do so is challenging.

The *Journal of Clinical and Translational Science* [5] and *Frontiers in Public Health* [6] have recently published thematic issues focused on the TSBM [7] for assessing research impact. The TSBM, developed at Washington University in St. Louis, is a structured framework that identifies benefits across four domains: clinical, community, economic, and policy. It also provides a process-oriented roadmap for prospectively planning and evaluating translational benefits across these domains throughout the life of a project.

This research letter illustrates how we applied TSBM to an ongoing research program involving clinical trials: *Heart Failure with Reduced Ejection Fraction (HFrEF) Polypill Implementation Trial in Sri Lanka*. Our aim is to highlight how the TSBM can be used to plan for, track, and communicate early and anticipated impacts during implementation, providing a clearer temporal context between ongoing and expected outcomes.

## Case example: HFrEF Polypill Implementation Trial in Sri Lanka

HFrEF is a leading global public health problem, with significantly higher mortality in low- and middle-income countries (LMIC) [8]. Despite high-quality evidence that guideline-directed medical therapy (GDMT) consisting of four classes of drugs substantially reduces the risk of mortality in patients with HFrEF, GDMT remains widely underused. A HFrEF polypill containing all four GDMT drugs in one pill may bridge this gap between evidence and clinical practice. The *HFrEF Polypill Implementation Trial in Sri Lanka* is currently in its early implementation and planning phase, encompassing pretrial mixed-methods research, a type I hybrid pilot randomized clinical trial (RCT), pilot trial process evaluation, and a forthcoming large-scale type I hybrid RCT to evaluate effectiveness, safety, and implementation of a HFrEF polypill-based implementation strategy in Sri Lanka [9]. Our application of TSBM focuses on early identification of benefits already observable – such as stakeholder coordination and regulatory engagement – and on potential future benefits that will evolve as the project progresses.

## Early integration of TSBM

**Figure 1.**
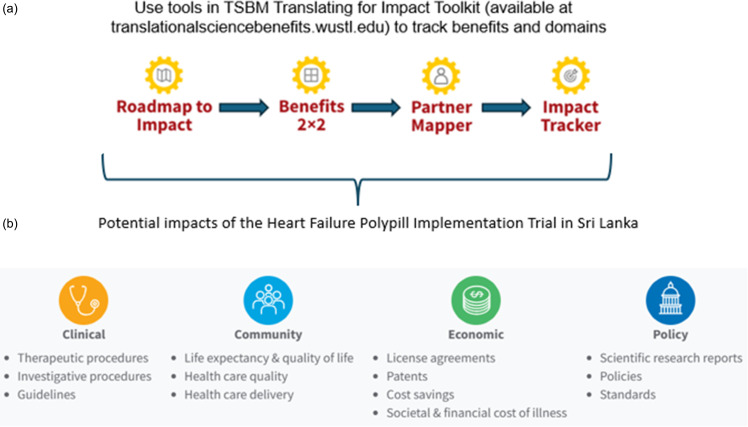
Planning tools and potential impacts assessed using the Translational Science Benefits Model.

To capture intermediary and projected impacts throughout the clinical trial, we embedded four tools from the free, online TSBM toolkit (www.translationalsciencebenefits.wustl.edu/toolkit/#/thetoolkit, Figure 1):

- **Roadmap to Impact**: Defines short, mid, and long-term milestones for patient outcomes, health system uptake, and policy integration.
- **Benefits 2 × 2**: Maps direct and indirect, short and long-term benefits to prioritize early metrics (e.g., feasibility, adherence, pill burden, and provider uptake) over longer-term endpoints like mortality or hospitalization.
- **Partner Mapper**: Identifies and engages stakeholders from clinical, governmental, and industry sectors relevant to the HFrEF polypill.
- **Impact Tracker**: Monitors progress toward potential adoption, including policy references and media engagement.

## Potential impacts

Clinical – Increase GDMT rates in patients with HFrEF by improving patient-level adherence and decrease provider-level clinical inertia.

Community – Enhance awareness and acceptance of simplified treatment strategies among patients, caregivers, and providers in South Asia.

Economic – Decrease in healthcare utilization and costs related to HF hospitalizations by reducing pill burden and improving adherence.

Policy – Inform national cardiovascular guidelines, support inclusion of HFrEF polypill on essential medicines lists, and inform procurement and reimbursement policies to expand access to HFrEF polypills if demonstrated to be safe and effective in clinical trials.

Each potential impact aligns with the domains represented in Figure 1, and early measurable indicators (e.g., adherence rates, dissemination reach, stakeholder participation) have been identified to track and refine progress over time.

## Why timing matters

Integrating impact assessment from the outset provides a framework that may help shape the strategic direction of the HFrEF Polypill program. Capturing and communicating impacts before primary research data are available has created opportunities to align with policymakers, foster partnerships with industry for scalable manufacturing, and build public awareness to facilitate adoption while interest is high. Waiting until research completion could delay these conversations and narrow the window for influence.

By integrating TSBM from the outset, clinical trials can evolve beyond traditional trial metrics to a multidimensional impact narrative that evolves alongside the science.

An iterative assessment process has been defined, consisting of quarterly project reviews using TSBM domains to evaluate progress, document stakeholder engagement, and identify opportunities for refinement.

Stakeholder engagement has been integral to implementation efforts. Partners include the Sri Lankan clinical research organization collaborators, healthcare providers, policymakers, and patients. Their feedback has informed feasibility measures, data collection priorities, and communication strategies for the ongoing trial.

Stakeholder feedback was solicited through a combination of semi-structured interviews, investigator meetings, and iterative review of study materials with implementation partners in Sri Lanka and the United States. Feedback was documented by the study team and used to refine feasibility metrics, data collection priorities, implementation workflows, and communication strategies.

## Conclusion

The *HFrEF Polypill Implementation Trial in Sri Lanka* offers one example of how TSBM can been used to capture translational benefits during a clinical trial research program. Applying the model has helped our team document and communicate early and projected impacts while engaging stakeholders proactively. TSBM’s prospective use provides a structured, iterative, and measurable approach to tracking translational impact across implementation stages.
